# Structure of *Vibrio* FliL, a New Stomatin-like Protein That Assists the Bacterial Flagellar Motor Function

**DOI:** 10.1128/mBio.00292-19

**Published:** 2019-03-19

**Authors:** Norihiro Takekawa, Miyu Isumi, Hiroyuki Terashima, Shiwei Zhu, Yuuki Nishino, Mayuko Sakuma, Seiji Kojima, Michio Homma, Katsumi Imada

**Affiliations:** a Department of Macromolecular Science, Graduate School of Science, Osaka University, Toyonaka, Osaka, Japan; b Division of Biological Science, Graduate School of Science, Nagoya University, Nagoya, Japan; c Department of Microbial Pathogenesis, Yale School of Medicine, Yale University, West Haven, Connecticut, USA; d Radioisotope Research Center, Nagoya University, Nagoya, Japan; University of Utah; University of Washington

**Keywords:** bacterial flagellar motor, crystal structure, mechanosensor, stator, stomatin

## Abstract

Many motile bacteria swim or swarm using a filamentous rotating organelle, the flagellum. FliL, a component protein of the flagellar motor, is known to enhance the motor performance under high-load conditions in some bacteria. Here we determined the structure of the periplasmic region of FliL (FliL_Peri_) of the polar flagellum of Vibrio alginolyticus. FliL_Peri_ shows a remarkable structural similarity to the stomatin/prohibitin/flotillin/HflK/C (SPFH) domain of stomatin family proteins, some of which are involved in modulation of ion channel activities in various organisms. FliL_Peri_ forms a ring assembly in the crystal with an inner diameter of around 8 nm, which is comparable to the size of the stator unit. Mutational analyses suggest that the FliL ring forms a complex with the stator unit and that the length of the periplasmic linkers of FliL and the stator B-subunit is essential for the complex formation. We propose a model of the FliL-stator complex to discuss how *Vibrio* FliL modulates stator function in the bacterial flagellar motor under conditions of high viscosity.

## Abstract (precis)

Some flagellated bacteria regulate motor torque in response to the external load change. This behavior is critical for survival, but the mechanism has remained unknown. Here, we focused on a key protein, FliL of Vibrio alginolyticus, and solved the crystal structure of its periplasmic region (FliL_Peri_). FliL_Peri_ reveals striking structural similarity to a conserved domain of stomatin, which is involved in ion channel regulation in some organisms, including mammals. FliL_Peri_ forms a ring with an inner diameter that is comparable in size to the stator unit. The mutational analyses suggested that the presence of the ring-like assembly of FliL around the stator unit enhances the surface swarming of *Vibrio* cells. Our study data also imply that the structural element for the ion channel regulation is conserved from bacteria to mammals.

## INTRODUCTION

Many bacteria move in physically distinct environments, such as soil, mucus, host cell surface, and planktonic environments, for survival in nature using the flagellum. The flagellum is one of the most common organelles for bacterial motility and consists of a filamentous propeller structure protruding from the cell surface and a rotational motor embedded in the cell envelope. To adjust their motility to the various physical environments, some bacteria, such as Escherichia coli and Bacillus subtilis, modify the flagellar motor power according to the external load (1–3). However, how they sense the external condition and optimize the motor output to the environment is not well understood.

The flagellar motor is made up of about 20 different types of proteins and is composed of two functionally distinct parts, the rotor and the stator. The motor is driven by electrochemical potential across the cell membrane. Coupling ion flow (e.g., H^+^, Na^+^, or K^+^ flow) through a channel in the stator induces the rotor-stator interaction to generate torque (4–6). The rotor consists of the rod, the MS-ring and the C-ring. The MS-ring is embedded in the cell membrane, and the C-ring is located beneath the MS-ring. The MS-ring is a homomultimer of a two-transmembrane protein, FliF. The C-ring is composed of three cytoplasmic soluble proteins, FliG, FliM, and FliN. The stator is a heterohexametric complex composed of four A-subunits and two B-subunits, PomA_4_PomB_2_ in the Na^+^-driven polar flagellar motor of Vibrio alginolyticus and MotA_4_MotB_2_ in the H^+^-driven flagellar motor of E. coli and *Salmonella* and the lateral flagellar motor of V. alginolyticus (7, 8). Up to about a dozen of stator units surround the rotor (9, 10).

The motor torque is generated by electrostatic interaction between FliG and the stator A-subunit (11). The stator B-subunit interacts with the peptidoglycan layer via the periplasmic region to anchor the stator unit (12, 13). The periplasmic region of the B-subunit contains a plug region that regulates the ion influx through the stator (14). The stator units are not static but dynamically assemble into and disassemble from the motor (11, 15). Although each single stator unit generates enough torque to rotate the filament under low-load conditions, a number of stators assemble around the rotor under high-load conditions to generate sufficient torque, suggesting that the flagellar motor somehow senses the load and adjusts its torque to the load by changing the number of stators around the rotor (1–3, 16, 17). A recent mutational study showed that the long cytoplasmic loop region of the A-subunit is responsible for the load-dependent assembly of the stator unit in *Salmonella* (18).

A flagellar protein, FliL, which is a single-transmembrane protein with a large periplasmic region and associates with the flagellar basal body, has been found to be important for the function of the motor under high-load conditions, such as the highly viscous environments in some species. FliL is essential for the swimming of Caulobacter crescentus and Rhodobacter sphaeroides and for the surface swarming of E. coli and *Salmonella*, whereas deletion of FliL does not strongly affect the swimming motility of E. coli, *Salmonella*, B. subtilis, Borrelia burgdorferi, Proteus mirabilis, and V. alginolyticus (19–26). A cryo-electron tomography analysis of B. burgdorferi showed that FliL is located close to the rotor and the stator (22). FliL interacts with various motor components, such as the stator proteins, the MS-ring protein FliF, and the periplasmic ring component FlgT (20, 21, 24, 27). Among them, the interaction with the stator seems to be most important for the function of FliL because some of the swimming defects caused by *fliL* mutations were suppressed by mutations in the plug region of the stator B-subunit (21, 24). Moreover, FliL did not assemble into the motor without the stator (25). Other mutational studies revealed various roles of FliL in addition to the motor function, including stabilization of the rod structure in the motor, correct positioning of the flagellum, and surface sensing (20, 22, 23, 28). However, most of the phenotypes of these mutants were diverse and species specific. Recently, a contradictory result has been reported that indicated that the torque of the E. coli motor is not changed by the deletion of FliL (29). Thus, the specific function of FliL is rather unclear.

In this study, we performed structural and mutational analyses of FliL to elucidate its molecular mechanism in V. alginolyticus. This bacterium has two different sets of flagella, i.e., a polar flagellum and multiple lateral flagella, and each set of the flagella has its own *fliL* gene. The polar flagellum is used for swimming in low-viscosity liquid (e.g., seawater), whereas the lateral flagella are used for surface swarming under conditions of very high loads (e.g., fish mucus) (30). Deletion of polar *fliL* did not strongly affect the swimming motility provided by polar flagellum under low-load conditions but reduced the swimming speed provided by polar flagellum in high-load conditions (25). On the other hand, the function of lateral FliL is unknown. Here we determined the structure of the periplasmic region of polar FliL (FliL_Peri_). FliL_Peri_ forms a ring or a helical assembly in crystal, and the subunit structure resembles the SPFH domain of the stomatin superfamily, which includes proteins involved in mechanosensory transduction and ion channel regulation, implying that FliL is involved in the regulation of the ion-channel activity of the stator in response to some kinds of mechanical signals. We also found that FliL is essential for the swarming provided by lateral flagellar motors. On the basis of the structure and the following mutational analyses, we propose a model for how FliL associates with the stator and regulates the flagellar motor function.

## RESULTS

### The length of the flexible linker of PomB is important for the FliL localization around the motor.

**FIG 1:**
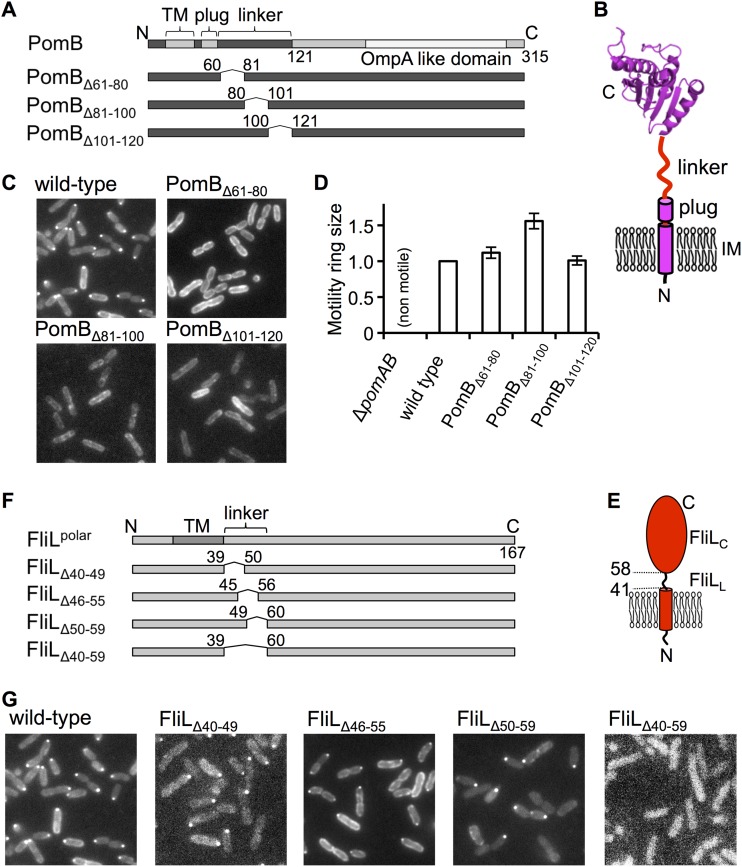
Effect of the in-frame deletion of the linker region of PomB and FliL on cell motility and FliL localization. (A) Molecular architecture of PomB and its in-frame deletion mutants. TM, transmembrane region. (B) Schematic drawing of the stator B subunit. The periplasmic domain of PomB (PDB ID: 3WPW) is represented in ribbon model form. IM, inner membrane. (C) Fluorescence images of cells expressing GFP-FliL with the in-frame deletion in PomB. (D) Effect of the in-frame deletions in PomB on the motility in a soft-agar plate. Motility ring diameters relative to that of the wild-type strain are shown. Error bars, standard deviations. Overnight cultures of ZSW2 harboring the plasmid pZSW81 with or without deletions in *pomB* were spotted onto VPG soft-agar plates and incubated at 30°C for 4 h. (E) Schematic drawing of FliL. (F) Molecular architecture of the in-frame linker deletion mutants of FliL. (G) Fluorescence images of cells expressing GFP-FliL with the in-frame deletions.

FliL interacts with the stator proteins and localizes at the motor. Deletion of the stator genes, i.e., *pomA* and *pomB*, completely disrupted the localization of polar FliL at the polar flagellar motor in V. alginolyticus (25). Here we introduced internal deletion mutations in the periplasmic flexible linker (residues 60 to 121) of PomB (Fig. 1A and B). It has been shown that the flexible linker region is not essential for the motor function but involved in motor efficiency (31). We prepared a series of in-frame deletion mutants which lacked 20 amino acid residues in the linker region and observed cell motility as well as the intracellular localization of polar FliL fused with green fluorescent protein (GFP) at its cytoplasmic N terminus (GFP-FliL) by fluorescence microscopy (Fig. 1C). As described previously, GFP-FliL was detected as a dot on the flagellated cell pole in the strain expressing wild-type PomB (Fig. 1C) (25). In contrast, GFP-FliL was not localized at the cell pole in all of the in-frame deletion mutants of PomB, even though these mutants showed swimming rings that were similar in size to those seen with the wild type in a soft-agar plate (Fig. 1D). These results indicate that the linker region of PomB is essential for FliL to localize at the motor and suggest that it is not a specific region but the length of the linker that is important for the FliL localization.

### The periplasmic region of FliL consists of the N-terminal linker and the C-terminal core region.

Polar FliL is composed of an N-terminal short cytoplasmic region (residues 1 to 17), a single transmembrane region (TM) (residues 18 to 40), and a periplasmic region (FliL_Peri_) (residues 41 to 167) (25). To determine the core region of FliL_Peri_, we performed limited proteolysis of purified FliL_Peri_ with trypsin. FliL_Peri_ was immediately digested into a fragment composed of residue 58 to 167 that is named FliL_C_, and FliL_C_ is highly resistant to trypsin digestion (see Fig. S1A to C in the supplemental material). Thus, we concluded that FliL_C_ is a core region of FliL_Peri_ and that the N-terminal 17 residues (residues 41 to 58; FliL_L_) act as a linker connecting the TM region with FliL_C_ (Fig. 1E). To evaluate the importance of FliL_L_ to the FliL function, we prepared mutants with various deletions in FliL_L_ and observed the FliL localization at the cell pole (Fig. 1F and G). FliL_Δ40-59_ did not localize at the cell pole, whereas FliL_Δ40-49_, FliL_Δ46-55_, and FliL_Δ50-59_ did. These results indicate that FliL_L_ is essential for the FliL localization but suggest that any 7 of the 17 residues are sufficient.

10.1128/mBio.00292-19.1 FIG S1 Limited proteolysis of the periplasmic region of FliL. Download FIG S1, PDF file, 0.2 MB. Copyright © 2019 Takekawa et al. 2019 Takekawa et al. This content is distributed under the terms of the Creative Commons Attribution 4.0 International license.

### Structure of the periplasmic region of FliL.

**FIG 2:**
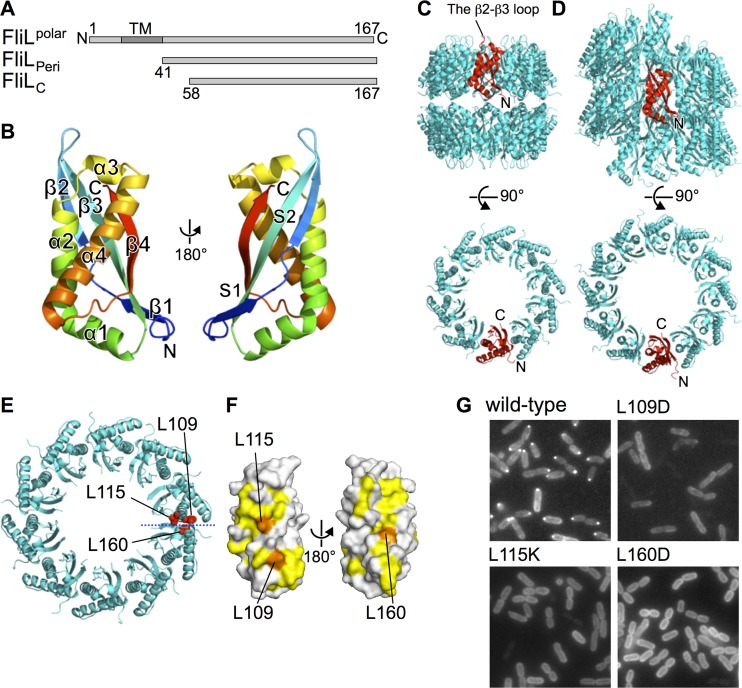
Structure of the periplasmic region of FliL. (A) Schematic representation of the molecular architecture of FliL, FliL_Peri_, and FliL_C_. (B) Ribbon drawing of FliL_58-167_ (FliL_C_) colored as a rainbow from the N terminus (blue) to the C terminus (red). (C and D) Higher-order structure of FliL_C_ in crystal. A ribbon representation of the two decameric rings in the *P*4_2_2_1_2 crystal (C) and the 12_1_ helical assembly in the *P*6_1_ crystal (D) are shown. A single subunit is colored red. (E) Mapping of the mutation sites that disrupt the FliL assembly. L109, L115, and L160 are indicated by red balls. (F) Cut-open view of the subunit interface indicated by the blue dashed line in panel E. L109, L115, and L160 are colored in orange, and other hydrophobic residues are colored in yellow. (G) Fluorescence images of cells expressing GFP-FliL or GFP-FliL with the L109D, L115K, or L160D mutation.

We crystallized and determined the structures of FliL_C_ and FliL_Peri_ at 2.1-Å and 3.4-Å resolution, respectively (Fig. 2A to D; see also Fig. S2A and B and Table S1 in the supplemental material). The initial phase was obtained by molecular replacement using a FliL_C_ structure predicted by the Robetta server (32), because no Se-Met-labeled crystals or heavy-atom-derivative crystals were obtained. The FliL_C_ crystal belongs to the space group of *P*6_1_ and contains two molecules in an asymmetric unit. The final refinement R factor and the free R factor values were 20.6% and 25.8%, respectively. FliL_C_ consists of a single domain with a bean shape containing four α-helices (α1 to α4) and one β-sheet composed of four β-strands (β1 to β4). The β-sheet is highly twisted at the middle and therefore can be divided in two subsheets, subsheet 1 (S1) and S2 (Fig. 2B). S1 consists of β1, β3, and β4, and S2 is composed of β2, β3, and β4. The FliL_Peri_ crystal belongs to the space group of *P*4_2_2_1_2 and contains 20 molecules in an asymmetric unit. FliL_Peri_ is 17 residues longer at its N terminus than FliL_C_, but these residues are invisible in the electron density map of FliL_Peri_. Therefore, we determined the structure of FliL_Peri_ with the same region as that of FliL_C_. All molecules in the asymmetric units of both FliL_C_ and FliL_Peri_ crystals showed no significant structural difference except for slight differences in the N-terminal region (residues 58 to 63) and in the loop between β2 and β3 (residues 77 to 84) (Fig. S2C).

10.1128/mBio.00292-19.2 FIG S2 Comparison of the FliL structures in crystal. Download FIG S2, PDF file, 0.2 MB. Copyright © 2019 Takekawa et al. 2019 Takekawa et al. This content is distributed under the terms of the Creative Commons Attribution 4.0 International license.

10.1128/mBio.00292-19.6 TABLE S1 Summary of the X-ray data statistics. Download Table S1, PDF file, 0.1 MB. Copyright © 2019 Takekawa et al. 2019 Takekawa et al. This content is distributed under the terms of the Creative Commons Attribution 4.0 International license.

### FliL_Peri_ forms a ring complex.

FliL_Peri_ forms a head-to-head stack of two decameric rings in the crystal asymmetric unit (Fig. 2C). The bean-shaped subunits are vertically arranged in a parallel manner in the ring. The two rings weakly interact with each other through α1. The N terminus locates near the ring-to-ring interface and points toward the outer side of the ring. The C terminus of each subunit is arranged on the inner surface of the ring on the opposite side of the ring-to-ring interface. The loop between β2 and β3 surrounding the hole of the ring contributes to the crystal contacts with the neighboring ring related by crystallographic symmetry. Since the N terminus of FliL_Peri_ is connected to the transmembrane helix, the decameric ring may be formed on the inner membrane (Fig. S2D).

The side-by-side subunit interaction in the ring is primarily hydrophobic. The subunit interface is formed by characteristic hydrophobic belts on both sides of the subunit along the longitudinal direction of the molecule (Fig. 2E and F; see also Fig. S2E). P108, L109, and L115 on α2 form a hydrophobic belt on one side of the subunit surface, and Y66 and M93 on subsheet S1 and L160, F161, and F164 on β4 form another on the opposite surface (Fig. 2F; see also Fig. S2E). L109, L115, and L160 contribute to the intersubunit mainly by hydrophobic interaction. L115 interacts with F161 and F164. L109 tightly interacts with L160 and slightly with M93 and Y66. The side-by-side interaction is further stabilized by hydrogen bonds around the hydrophobic belts (Fig. S2E). N76 forms a hydrogen bond with the main-chain NH group of I166. Q71, Y105, and D163 form hydrogen bonds with the main-chain carbonyl group consisting of T162, I64, and V74, respectively. The side chain of H106 forms hydrogen bonds with the OH group of Y66. Similar subunit interactions can be observed in the FliL_C_ crystal. The two molecules in the asymmetric unit of the FliL_C_ crystal are related by 30° rotation around the crystallographic 6_1_ screw axis with a translation of 1/12 of the repeat along the *c* axis; thus, the molecules form a helical tube with 12_1_ screw symmetry along the *c* axis in the FliL_C_ crystal (Fig. 2D). The interaction between the subunits related by the 12_1_ screw symmetry is almost the same as that in the FliL_Peri_ ring except for the lack of hydrogen bonds between T162 and Q71 and between D163 and V74. The neighboring subunit in the FliL_C_ crystal is slightly tilted compared with that in the FliL_Peri_ ring, resulting in the formation of the helical tube rather than the ring (Fig. S2F).

The common side-by-side subunit interaction implies that assembly formation through the hydrophobic belt is important for the FliL function. To analyze the significance of the hydrophobic interaction, we replaced the residues that mainly contribute to the hydrophobic interaction with hydrophilic residues (L109D, L115K, or L160D) and observed the localization of GFP-FliL (Fig. 2E to G). The polar localization of GFP-FliL was completely disrupted by these mutations. Other mutations of the residues on the outer, top, and bottom surfaces of the FliL ring did not affect the polar localization of FliL (Fig. S3A and B). We further confirmed that FliL interacts with itself without any help from other flagellar proteins in the cell by two-hybrid assay (Fig. S3C). These results indicate that the FliL ring formation by the hydrophobic interaction is essential for the FliL assembly into the motor.

10.1128/mBio.00292-19.3 FIG S3 Fluorescence images of cells expressing various FliL mutant proteins labeled with GFP and results of bacterial two-hybrid assay. Download FIG S3, PDF file, 0.2 MB. Copyright © 2019 Takekawa et al. 2019 Takekawa et al. This content is distributed under the terms of the Creative Commons Attribution 4.0 International license.

### The FliL_C_ structure resembles the SPFH domain of stomatin.

**FIG 3:**
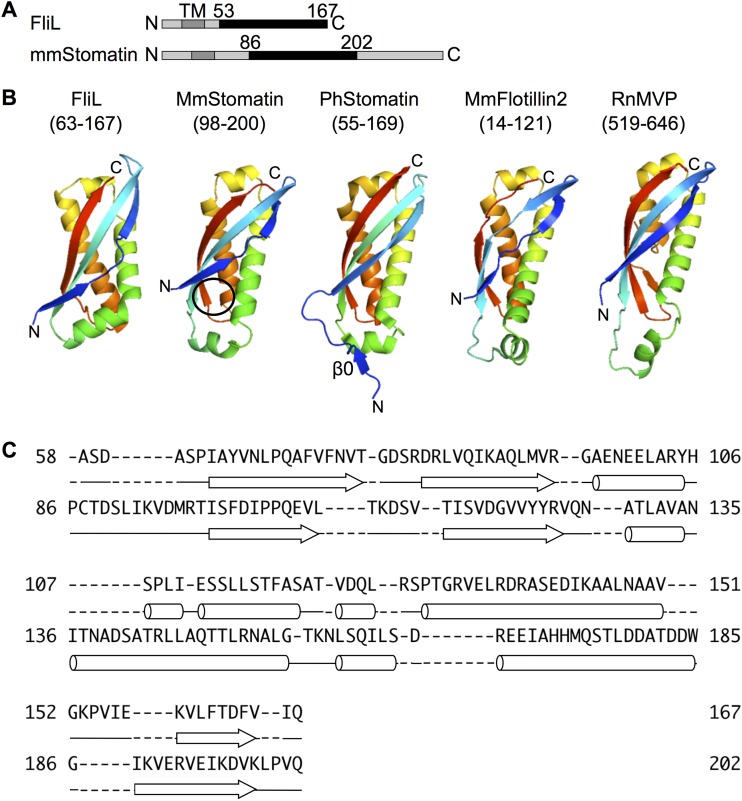
Comparison of FliL with the SPFH domain. (A) Molecular architecture of FliL and Mus musculus stomatin (MmStomatin). The transmembrane regions (TM) are indicated in dark gray. FliL_C_ and the SPFH domain are shown in black. (B) Ribbon drawing of FliL_C_ (amino acids 63 to 167), of the SPFH domain of MmStomatin (amino acids 98 to 200) (PDB ID: 4FVJ), of Pyrococcus horikoshii stamatin (PhStomatin; amino acids 55 to 169) (PDB ID: 3BK6), of Mus musculus flotillin-2 (MmFlotillin; amino acids 14 to 121) (PDB ID: 1WIN), and of Rattus norvegicus major vault protein (RnMVP; amino acids 519 to 646) (PDB ID: 4V60). The hydrophobic surface interacting with the N-terminal loop of the neighboring subunit in the MmStomatin multimer is shown by a black circle. (C) Structure-based sequence alignment of FliL_C_ (the upper sequence) and the SPFH domain of Mm-stomatin (the lower sequence). Cylinder, α-helix; arrow, β-strand.

The Dali database search revealed that FliL_C_ shows remarkable structural similarity (Z-score of 9.5) to the stomatin/prohibitin/flotillin/HflK/C (SPFH) domain of stomatin from Mus musculus (Mm-stomatin) (Fig. 3A and B) (PDB identifier [ID]: 4FVJ) (33), although the amino acid sequence identity is only 17% (Fig. 3C). The SPFH domain is conserved in various membrane-associated proteins of eukaryotes, archaea, and bacteria, such as stomatin, prohibitin, flotillin, HflK/C, and podocin (34). The SPFH domain is composed of four alpha helices and a highly twisted β-sheet, and its folding topology is exactly the same as that of FliL_C_ (Fig. 3B and C).

The SPFH domain is often involved in multimer formation of the SPFH superfamily proteins. In spite of the structural similarity, the subunit arrangement and the subunit interface in the FliL_C_ multimer differ from those in the SPFH family protein multimer. The Mm-stomatin assembles into a tail-to-tail dimer by forming an intersubunit β-sheet via the C-terminal β-strands (Fig. S4A) (33). However, FliL_C_ does not form the tail-to-tail dimer. The Mm-stomatin dimers form a helical higher-order assembly in the crystal through the interaction between the N-terminal loop and the hydrophobic surface of the neighboring subunit. Although FliL_C_ also forms a helical assembly in the *P*6_1_ crystal, the FliL_C_ assembly is formed by the lateral subunit interaction and the corresponding hydrophobic surface of FliL_C_ is not accessible because it is covered by the N-terminal β-strand (Fig. 3B). The stomatin from Pyrococcus horikoshii (Ph-stomatin) assembles into a trimer formed by head-to-tail interaction of the unique N-terminal β-strand, β0, with the C-terminal β-strand of the neighboring subunit (Fig. S4B) (35). However, FliL_C_ does not form a trimer and does not have the counterpart of β0. The SPFH domains of the major vault protein are laterally arranged in the vault particle in a manner similar to that seen with the FliL_C_ subunits in the ring structure, but the interaction surface to the neighboring subunit is different (Fig. S4C) (36). Therefore, the positions of the neighboring molecules differ by about 60 degrees.

10.1128/mBio.00292-19.4 FIG S4 Multimer of the SPFH domains. Download FIG S4, PDF file, 0.2 MB. Copyright © 2019 Takekawa et al. 2019 Takekawa et al. This content is distributed under the terms of the Creative Commons Attribution 4.0 International license.

## DISCUSSION

The flexible linker of the stator B-subunit following the plug region is not essential for the motor rotation (31) but is necessary to control the number of active stator units around the rotor in response to the external load (3, 16). Since the linker in PomB is required for the FliL localization at the motor, FliL is expected to directly interact with the PomB linker region to regulate the motor output. On the other hand, all of the mutants with 20-residue deletions in various regions of the PomB linker lost the FliL localization ability at the motor. This suggests that FliL does not interact with a specific region of the PomB linker and that the length of the linker is important for the interaction with FliL. FliL also has a flexible linker (FliL_L_) that is essential for the FliL localization. FliL_L_ connects the transmembrane helix with FliL_C_. The analysis of various deletion mutants in FliL_L_ suggests that it is not a specific region in FliL_L_ but the length of the linker that is critical for the FliL localization, as in the case of the PomB linker. It is probable that the positional flexibility of the periplasmic domains of PomB and FliL attributed to the linker regions with appropriate length is needed for the PomB-FliL interaction.

We have determined the structure of the periplasmic core region of polar FliL (FliL_C_). FliL_C_ shows remarkable structural similarity to the SPFH domain despite the low similarity of amino acid sequence (Fig. 3B and C). Although the physiological function of the SPFH domain has not yet been well characterized, some of the SPFH family proteins are known to interact with various ion channels and transporters to modulate their activities (33, 37, 38). These proteins are involved in mechanosensory transduction in mammalian sensory neurons and in mechanosensor formation in Caenorhabditis elegans (39, 40), but the mechanosensing mechanism is still unknown (41). The structural similarity between FliL_C_ and the SPFH domain implies that FliL is involved in mechanosensing by the flagellum and in regulation of the channel activity of the stator. In fact, FliL of P. mirabilis is involved in the mechanosensing pathway (23, 28). It is possible that FliL and these SPFH family proteins share a mechanism for mechanosensing and mechanoresponse.

The periplasmic domain of FliL forms a decameric ring or a helical tube complex with 12_1_ screw symmetry in the crystals, and the two complexes share similar lateral subunit interactions. The mutations of the hydrophobic residues on the subunit interface to hydrophilic ones disrupted the FliL localization at the motor. This suggests that FliL forms a ring-like oligomer on the cell membrane (see Fig. S2D in the supplemental material) and that the oligomerization is essential for the function of FliL. The idea of the assembly formation of FliL is also supported by the results of two-hybrid assays (Fig. S3C). Moreover, it is consistent with our previous reports of findings indicating that the periplasmic region of FliL oligomerizes at high concentrations (42). Similar ring structures have been reported in the SPFH family proteins, such as human stomatin and mitochondrial prohibitin of Saccharomyces cerevisiae (43, 44). They form ring complexes composed of 9 to 12 subunits on the cell membrane. On the other hand, some other SPFH family proteins whose structures are known have shown diverse assembly states and subunit arrangements in multimer (Fig. S4). Mm-stomatin assembles into a tubular complex composed of antiparallel dimers (Fig. S4A) (33), and Ph-stomatin forms a triangular complex of three molecules (Fig. S4B) (35). The SPFH domain of the rat liver vault forms a 39-fold ring through the lateral interaction in the vault (Fig. S4C) (36). The subunit interactions of FliL_C_ completely differ in those SPFH domains (Fig. 2C and D). The SPFH domains do not have the hydrophobic belts on the interface, which is important for the ring formation of FliL (Fig. S4D). The subunit arrangement and the interaction manner of the SPFH domains may be optimized for their individual function and/or their regulation partner proteins.

**FIG 4:**
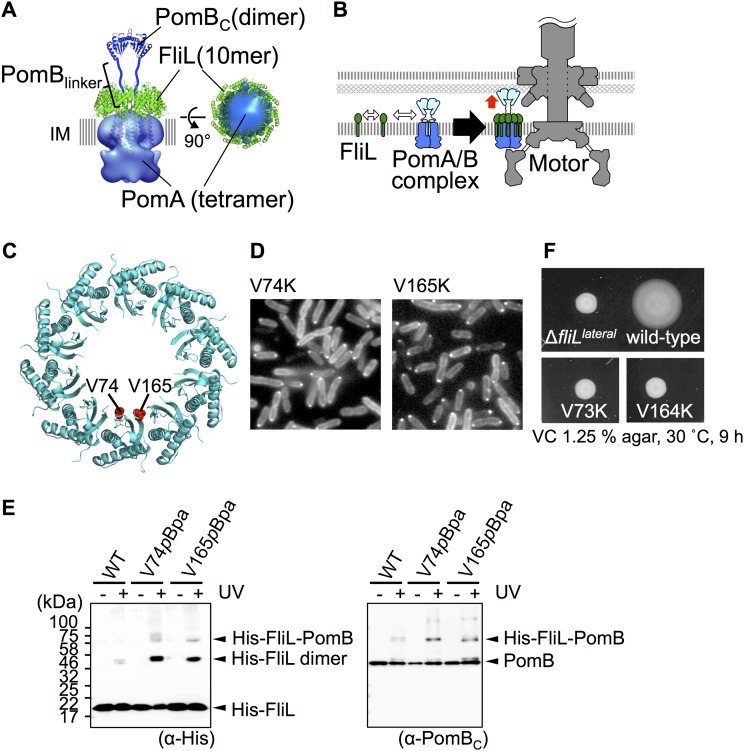
A plausible model for the assembly and function of FliL (A) A FliL-stator complex model. The ring structure of FliL_Peri_ is fitted to the stator model constructed by the electron-microscopic structure of MotA (a PomA orthologue) (EMDB ID: 3417) and the crystal structure of periplasmic domain of PomB (PomB_C_) (PDB ID: 3WPW). IM, inner membrane. (B) A model for the assembly of FliL. The white two-way arrows indicate the protein-protein interactions, and the red arrow indicates the activation of the stator. (C) The positions of conserved valine residues (V74 and V165) in the FliL ring. (D) Fluorescence images of cells expressing GFP-FliL with the mutations at conserved valine. (E) *In vivo* photo-cross-linking between FliL and PomB. WT, wild type. (F) Effect of the mutations at the conserved valine on surface swarming by lateral flagella. The lateral FliL proteins with or without mutations were expressed from pZSW91 in NMB342. An overnight culture was spotted onto VC hard-agar plates containing 0.02% (wt/vol) arabinose and incubated at 30°C for 9 h.

FliL modulates the motor torque by direct interaction with stator proteins in E. coli and *Salmonella* (24) and requires the stator to localize at the motor in V. alginolyticus (25). A motility defect in R. sphaeroides caused by the *fliL* mutation was suppressed by mutation in the plug region of MotB (21). These lines of evidence suggest that FliL forms a complex with the stator. Our crystal structure revealed that FliL_C_ forms a ring with an inner diameter of around 8 nm (Fig. 2C), which is a size comparable to that of the stator unit. We then built a FliL-stator complex model by fitting the FliL ring structure to the stator model constructed by the electron microscopy (EM) density of MotA at 25-Å resolution (EMDB ID: 3417) (45) and the crystal structure of the periplasmic domain of PomB (PDB ID: 3WPW) (46). The stator was nicely accommodated within the FliL ring (Fig. 4A), and the plug region of the stator B-subunit locateds in close proximity to the inner surface of the FliL ring (Fig. 4B).

This model is supported by results of a mutation analysis of the residues on the inner surface of the ring (Fig. 4C to F). We replaced conserved valine residues on the inner surface of the FliL ring (V74 and V165) with hydrophilic ones and analyzed the FliL localization. These mutations did not affect the GFP-FliL localization (Fig. 4D). We next replaced these valine residues with *p*-benzoyl-l-phenylalanine (*p*Bpa), which is a photoreactive unnatural amino acid and forms a covalent bond with nearby protein molecules upon UV irradiation. The mutated FliL protein was coexpressed with V. alginolyticus PomA/PomB proteins in E. coli BL21(DE3) cells, and UV light was used for irradiation of the cells for photo-cross-linking. The mutant FliL was cross-linked to PomB by the use of UV light (Fig. 4E), indicating that V74 and V165 of FliL are close to PomB *in vivo*. Since E. coli BL21(DE3) cells have no flagellum, this result suggests that FliL can assemble around the stator without the flagellar basal body. We further examined the effect of a corresponding mutation of lateral FliL on the surface motility under high-load conditions. Deletion of lateral *fliL* in the YM19 strain, which forms only lateral flagella, resulted in loss of swarming ability on a 1.25% agar plate (Fig. 4F; see also Fig. S5A) but did not strongly affect swimming in a 0.25% agar plate (Fig. S5A). We confirmed that lateral flagellar formation was not affected by the deletion of *fliL* in the wild-type strain (strain 138-2) by electron microscopy and Western blotting (Fig. S5B and C). The replacement of the corresponding valine residues of lateral FliL (V73 and V164) with the hydrophilic ones disrupted the swarming motility by lateral flagella (Fig. 4F). These results suggest that the conserved valine residues, located at the inner side of the FliL ring, are not essential for the complex formation with the stator but are close to PomB and important for activation of the stator in the swarming cells.

10.1128/mBio.00292-19.5 FIG S5 Effect of mutations on the motility and flagellation. Download FIG S5, PDF file, 0.2 MB. Copyright © 2019 Takekawa et al. 2019 Takekawa et al. This content is distributed under the terms of the Creative Commons Attribution 4.0 International license.

A recent fluorescence microscopic analysis using FliL fused with GFP at its N terminus (GFP-FliL) suggested that the stoichiometry of a stator unit and FliL protein in a functional motor would be 1:1 (47), which is inconsistent with the model presented in this study. We found, however, that GFP-FliL impairs the motor function (Fig. S5D). The mutant cells expressing GFP-FliL reduced the motility less than the Δ*fliL* mutant cells. This motility reduction was suppressed by the L109D mutation or the L160D mutation, each of which disrupts the localization of FliL (Fig. S5D). It is possible that the number of stators around the motor is reduced by the presence of GFP. Therefore, it would be difficult to estimate the number of FliL proteins in a functional motor using GFP-FliL.

A previous cryo-electron tomographic study of an *in situ* flagellar basal body of B. burgdorferi resulted in a report that FliL is located between the stator and the rotor (22). However, the stator unit of B. burgdorferi is flanked by a collar, which is a basal-body component specific for spirochetes. Therefore, we cannot directly compare the *Borrelia* basal body structure with that of *Vibrio*, but another extra density is present on the outer side of the stator density in their tomogram (22) which is consistent with our ring model.

On the basis of our results together with the previous reports, we propose a model for the regulation mechanism of motor output by FliL (Fig. 4B). The FliL molecules may surround the stator to form the FliL-stator complex at the motor. The conformational flexibility attributed to the linker regions of FliL and PomB may be important to adjust each subunit in the appropriate position to form the FliL-stator complex. The inner-surface residues of the FliL_C_ ring may then interact with the periplasmic region of PomB, presumably representing the plug region that is present to keep the ion influx, to increase the motor torque, especially under swarming conditions. The structural similarity to the SPFH domains of the stomatin family proteins implies that FliL may regulate the ion channel activity of the stator in response to some mechanical signals. However, mechanosensing mechanism is still unknown for all stomatin family proteins. Another possible mechanism of regulation of motor output is that the FliL ring acts as the catch-bond proposed by Nord et al. (17). FliL may assist the binding of the B-subunit to the peptidoglycan layer under high-load conditions and may thus prevent dissociation of the stator unit from the motor. Further studies are required to clarify the regulation mechanism of motor output in response to external load as well as the load-sensing mechanism.

## MATERIALS AND METHODS

### Bacterial strains, media, and plasmids.

The bacterial strains and plasmids used in this study are listed in Table S2 in the supplemental material. V. alginolyticus was cultured at 30°C in VC medium (0.5% [wt/vol] polypeptone, 0.5% [wt/vol] yeast extract, 0.4% [wt/vol] K_2_HPO_4_, 3% [wt/vol] NaCl, 0.2% [wt/vol] glucose) or VPG medium (1% [wt/vol] polypeptone, 0.4% [wt/vol] K_2_HPO_4_, 3% [wt/vol] NaCl, 0.5% [wt/vol] glycerol). If needed, chloramphenicol was added at a final concentration of 2.5 μg ml^−1^ for *Vibrio* culture. E. coli was cultured in LB medium (Lennox; Nacalai Tesque, Kyoto, Japan). If needed, chloramphenicol or ampicillin was added at a final concentration of 25 μg ml^−1^ or 50 μg ml^−1^ for E. coli culture.

10.1128/mBio.00292-19.7 TABLE S2 Strains and plasmids used in this study. Download Table S2, PDF file, 0.1 MB. Copyright © 2019 Takekawa et al. 2019 Takekawa et al. This content is distributed under the terms of the Creative Commons Attribution 4.0 International license.

To construct the plasmids, PCR-amplified DNA fragments were cloned into each plasmid vector by using T4 DNA ligase (New England Biolabs) or *in vivo* cloning (48).

To delete the *fliL* genes in V. alginolyticus, the upstream sequence (ca. 500 bp) and downstream sequence (ca. 500 bp) of the *fliL* gene were cloned into pSW7848 and homologous recombination was performed as previously described (49). Point mutations and internal deletion mutations in the plasmids were introduced by QuikChange site-directed mutagenesis (Agilent Technologies).

Transformation of E. coli was performed using a standard heat shock method. Transformation of V. alginolyticus was performed by the use of the electroporation method described below. Overnight cultures grown in VC medium were inoculated at a 100-fold dilution into 5 ml of VC medium and cultivated for 90 min at 30°C. The cells were harvested and suspended in 1 ml of ice-cold SNT buffer (30 mM Tris-HCI [pH 8.0], 20% [wt/vol] sucrose, 0.4 M NaCl, 1 mM EDTA), followed by gentle shaking for 10 min at 4°C. The cells were precipitated and washed by 1 ml of ice-cold SP buffer (14% [wt/vol] sucrose, 3.5 mM K_2_HPO_4_, 3.5 mM NaH_2_PO_4_). The cells were precipitated again, suspended in 40 μl of ice-cold SP buffer containing plasmid, and transferred into a 0.1-cm-gap electroporation cuvette. Electroporation was carried out with a gene pulser (MicroPulse Electroporator; Bio-Rad Laboratories, Hercules, CA, USA) at 7 kV cm^−1^ of electric field strength. After electroporation, 1 ml of VC medium was added immediately and incubated at 30°C for 45 min. The cells were precipitated, spread on a selective plate containing antibiotics, and incubated overnight at 30°C.

### Soft-agar and hard-agar plate assays for motility.

A 2-µl volume of overnight culture was spotted onto VPG soft-agar plates (VPG containing 0.25% [wt/vol] agar) or VC hard-agar plates (VC containing 1.25% [wt/vol] agar) followed by incubation at 30°C for various time periods as described in the text. Motility ring diameters were measured relative to those of the wild-type cells. The averaged values and standard deviations of data from more than three independent experiments were plotted in the graphs.

### Observation of subcellular localization of GFP-FliL with fluorescence microscopy.

Overnight cultures grown in VC medium were inoculated at a 100-fold dilution into VPG medium containing 0.006% (wt/vol) arabinose and cultivated for 4 h at 30°C. Cells were harvested by centrifugation and resuspended in TMN500 buffer (50 mM Tris-HCl [pH 7.5], 5 mM MgCl_2_, 5 mM glucose, 500 mM NaCl). The cells were then loaded into the space between a coverslip and a microscope slide, both of which are covered with poly-l-lysine, and incubated for 10 min for attachment to the coverslip surface. After washing away of unbound cells by the use of TMN500 buffer, the cells were observed by the use of a fluorescence microscope (BX53; Olympus, Japan) equipped with a 100-W high-pressure mercury lamp (U-RFL-T; Olympus). Images were recorded using a digital charge-coupled-device (CCD) camera and an acquisition program (INFINITY2-1RM and INFINITY CAPTURE; Argo Corporation, Japan). Strain ZSW1 harboring pZSW6 was used for the observation of the FliL mutant cells, and strain ZSW2 harboring pZSW81 was used for the observation of the PomB mutant cells.

### Purification of periplasmic fragments of FliL.

Expression and purification of FliL_Peri_ were performed as previously described (42). FliL_C_ was prepared from FliL_Peri_ by limited proteolysis (50). His_6_-FliL_Peri_, purified by nickel-nitrilotriacetic acid (Ni-NTA) affinity chromatography, was digested using trypsin at the protease/protein ratio of 1/100 (wt/wt) for 30 min at 27°C, and the products were separated by size exclusion chromatography (Superdex 75 10/300 GL; GE Healthcare, Chicago, IL, USA).

### Crystallization and structure determination.

Crystallization was carried out using the sitting-drop vapor-diffusion method. Details of the crystallization screening were previously described (42). FliL_Peri_ crystals used for X-ray data collection were grown at 20°C from drops prepared by mixing 0.5 to 1 μl protein solution (70 mg ml^−1^) containing 20 mM Tris-HCl (pH 8.0) and 150 mM NaCl with the equivalent volume of reservoir solution containing 0.1 M sodium acetate (pH 6.0), 25% (vol/vol) polyethylene glycol (PEG) 200, 0.15 M CaCl_2_ and 1.4% (vol/vol) ethyl alcohol (EtOH). The crystals belong to the space group of *P*4_2_2_1_2 with unit cell dimensions of *a *=* b *=* *162.2 and *c *=* *302.2 Å. The FliL_C_ crystals used for X-ray data collection were obtained at 20°C from drops prepared by mixing 0.5 to 1 μl protein solution (10 mg ml^−1^) containing 20 mM Tris-HCl (pH 8.0) and 150 mM NaCl with the equivalent volume of reservoir solution containing 0.1 M sodium acetate (pH 4.4), 22.5% (wt/vol) PEG 4000, and 0.2 M ammonium sulfate. The crystals belong to the space group of *P*6_1_ with unit cell dimensions of *a *=* b *=* *104.2 and *c *=* *40.1 Å.

X-ray diffraction data were collected at synchrotron beamline BL41XU in SPring-8 (Harima, Japan) with the approval of the Japan Synchrotron Radiation Research Institute (JASRI) (Proposal no. 2015A1084, 2015B1084, 2016A2541, 2016B2541, 2017A2588, and 2017B2588). Crystals were frozen in liquid nitrogen and mounted in nitrogen gas flow at 100 K for X-ray data collection. The diffraction data were processed with MOSFLM (51) and were scaled with AIMLESS (52). The statistics of the diffraction data are summarized in Table S1.

The initial phase of the FliL_C_ crystal was calculated using the molecular replacement method with Phenix software (53) and a predicted FliL_C_ structure model produced by the Robetta server (http://robetta.bakerlab.org/) (32) as a search model. The atomic model of FliL_C_ was built with Coot (54) and refined to 2.1-Å resolution with Phenix (53). The structure of FliL_Peri_ was determined by molecular replacement with Phenix using the atomic model of FliL_C_ as a search model. The model was modified with Coot (54) and refined to 3.4-Å resolution with Phenix. The refinement statistics are summarized in Table S1.

### *In vivo* photo-cross-linking.

BL21(DE3) cells carrying two different plasmids, pEVOL-*p*BpF and pNT70 with or without the V74amber or V165amber mutation, were cultured in LB medium containing 1 mM *p*-benzoyl-l-phenylalanine (*p*Bpa) (Bachem AG, Switzerland) for 2.5 h at 30°C from an initial optical density of 600 nm of 0.1. Then, arabinose and isopropyl-β-d-thiogalactopyranoside (IPTG) were added to reach final concentrations of 0.02% (wt/vol) and 0.1 mM, respectively, and the cultivation was continued for 4 h. Cells were collected by centrifugation (3,000 × *g*), suspended in PBS buffer (137 mM NaCl, 2.7 mM KCl, 10 mM Na_2_HPO_4_, 1.76 mM KH_2_PO_4_), collected again by centrifugation, and resuspended in PBS buffer. The UV irradiation was performed with B-100AP UV lamp (Analytik Jena US, Upland, CA, USA) for 5 min. The cells were then disrupted by sonication and centrifuged at 15,000 × *g* for 5 min. The supernatant was ultracentrifuged at 100,000 × *g* for 30 min. The precipitant was suspended in TN buffer (20 mM Tris-HCl [pH 8.0], 150 mM NaCl) containing 1% (wt/vol) n-dodecyl-β-d-maltopyranoside (DDM) and incubated at 4°C for 1 h. The solubilized membrane fraction was mixed with Ni-NTA agarose (Qiagen, Hilden, Germany) at 4°C for 1 h. The protein-bound agarose was washed twice with TN buffer containing 0.1% (wt/vol) DDM and 10 mM imidazole, and the proteins were subsequently eluted with TN buffer containing 0.1% (wt/vol) DDM and 300 mM imidazole. The proteins were precipitated by trichloroacetic acid precipitation and suspended in sodium dodecyl sulfate (SDS) loading buffer (62.5 mM Tris-HCl [pH 6.8], 2% [wt/vol] SDS, 10% [wt/vol] glycerol, 0.01% [wt/vol] bromophenol blue) containing 5% (vol/vol) β-mercaptoethanol and were boiled at 95°C for 3 min. Samples were separated by SDS-polyacrylamide gel electrophoresis (SDS-PAGE) and transferred to a polyvinylidene difluoride membrane. The proteins were detected using mouse anti-His antibody and rabbit anti-PomB_C_ antibody (55).

### Bacterial two-hybrid assay.

We used the bacterial two-hybrid system developed by Karimova et al. (56). DHM1 cells, carrying the plasmids derived from pKT25 and pUT18C, were grown in LB medium containing 50 μg ml^−1^ ampicillin and 50 μg ml^−1^ kanamycin at 37°C for about 24 h, and cell cultures were spotted onto a BacTH plate (LB containing 1.25% [wt/vol] agar, 0.5 mM IPTG [isopropyl-β-d-thiogalactopyranoside], 40 μg ml^−1^ 5-bromo-4-chloro-3-indolyl-β-d-galactopyranoside [X-Gal], 50 μg ml^−1^ ampicillin, and 50 μg ml^−1^ kanamycin) and incubated at 30°C for about 24 h.

### Transmission electron microscopy.

The cells of V. alginolyticus strain NMB338 or strain NMB339 were picked from fresh colonies on the agar plates and mixed with 2% (wt/vol) potassium phosphotungstate solution (pH 7). The solution was placed on a carbon-coated copper grid and observed with an electron microscope (JEM-2010; JEOL, Japan).

### Detection of lateral flagella by immunoblotting.

Overnight cultures grown in VC medium were inoculated into VPG medium at a 100-fold dilution and were cultivated at 30°C for 4 h. Cells were harvested by centrifugation, suspended to an optical density at 660 nm of 10 in SDS loading buffer containing 5% (vol/vol) β-mercaptoethanol, and boiled at 95°C for 5 min. Samples were separated by SDS-PAGE and transferred to a nitrocellulose membrane. The proteins were detected using rabbit anti-lateral flagellum polyclonal antibody raised against the purified lateral flagellin.

### Data availability.

The atomic coordinates have been deposited in the Protein Data Bank (www.pdb.org) (PDB ID: 6AHP and 6AHQ).

## References

[1] Lele PP, Hosu BG, Berg HC 2013 Dynamics of mechanosensing in the bacterial flagellar motor. Proc Natl Acad Sci U S A 110:11839–11844. doi:10.1073/pnas.1305885110. 23818629 PMC3718179

[2] Tipping MJ, Delalez NJ, Lim R, Berry RM, Armitage JP 2013 Load-dependent assembly of the bacterial flagellar motor. mBio 4:e00551-13. doi:10.1128/mBio.00551-13. 23963182 PMC3747592

[3] Terahara N, Noguchi Y, Nakamura S, Kami-Ike N, Ito M, Namba K, Minamino T 2017 Load- and polysaccharide-dependent activation of the Na^+^-type MotPS stator in the *Bacillus subtilis* flagellar motor. Sci Rep 7:46081. doi:10.1038/srep46081. 28378843 PMC5380961

[4] Berg HC 2003 The rotary motor of bacterial flagella. Annu Rev Biochem 72:19–54. doi:10.1146/annurev.biochem.72.121801.161737. 12500982

[5] Minamino T, Imada K 2015 The bacterial flagellar motor and its structural diversity. Trends Microbiol 23:267–274. doi:10.1016/j.tim.2014.12.011. 25613993

[6] Ito M, Takahashi Y 2017 Nonconventional cation-coupled flagellar motors derived from the alkaliphilic *Bacillus* and *Paenibacillus* species. Extremophiles 21:3–14. doi:10.1007/s00792-016-0886-y. 27771767

[7] Sato K, Homma M 2000 Multimeric structure of PomA, the Na^+^-driven polar flagellar motor component of *Vibrio alginolyticus*. J Biol Chem 275:20223–20228. doi:10.1074/jbc.M002236200. 10783392

[8] Braun TF, Al-Mawsawi LQ, Kojima S, Blair DF 2004 Arrangement of core membrane segments in the MotA/MotB proton-channel complex of *Escherichia coli*. Biochemistry 43:35–45. doi:10.1021/bi035406d. 14705929

[9] Leake MC, Chandler JH, Wadhams GH, Bai F, Berry RM, Armitage JP 2006 Stoichiometry and turnover in single, functioning membrane protein complexes. Nature 443:355–358. doi:10.1038/nature05135. 16971952

[10] Reid SW, Leake MC, Chandler JH, Lo CJ, Armitage JP, Berry RM 2006 The maximum number of torque-generating units in the flagellar motor of *Escherichia coli* is at least 11. Proc Natl Acad Sci U S A 103:8066–8071. doi:10.1073/pnas.0509932103. 16698936 PMC1472430

[11] Zhou JD, Lloyd SA, Blair DF 1998 Electrostatic interactions between rotor and stator in the bacterial flagellar motor. Proc Natl Acad Sci U S A 95:6436–6441. doi:10.1073/pnas.95.11.6436. 9600984 PMC27776

[12] De Mot R, Vanderleyden J 1994 The C-terminal sequence conservation between OmpA-related outer membrane proteins and MotB suggests a common function in both gram- positive and gram-negative bacteria, possibly in the interaction of these domains with peptidoglycan. Mol Microbiol 12:333–334. doi:10.1111/j.1365-2958.1994.tb01021.x. 8057857

[13] Kojima S, Takao M, Almira G, Kawahara I, Sakuma M, Homma M, Kojima C, Imada K 2018 The helix rearrangement in the periplasmic domain of the flagellar stator B subunit activates peptidoglycan binding and ion influx. Structure 26:590–598. doi:10.1016/j.str.2018.02.016. 29576320

[14] Hosking ER, Vogt C, Bakker EP, Manson MD 2006 The *Escherichia coli* MotAB proton channel unplugged. J Mol Biol 364:921–937. doi:10.1016/j.jmb.2006.09.035. 17052729

[15] Fukuoka H, Wada T, Kojima S, Ishijima A, Homma M 2009 Sodium-dependent dynamic assembly of membrane complexes in sodium-driven flagellar motors. Mol Microbiol 71:825–835. doi:10.1111/j.1365-2958.2008.06569.x. 19183284

[16] Castillo DJ, Nakamura S, Morimoto YV, Che Y-S, Kami-Ike N, Kudo S, Minamino T, Namba K 2013 The C-terminal periplasmic domain of MotB is responsible for load-dependent control of the number of stators of the bacterial flagellar motor. Biophysics (Nagoya-Shi) 9:173–181. doi:10.2142/biophysics.9.173. 27493556 PMC4629673

[17] Nord AL, Gachon E, Perez-Carrasco R, Nirody JA, Barducci A, Berry RM, Pedaci F 2017 Catch bond drives stator mechanosensitivity in the bacterial flagellar motor. Proc Natl Acad Sci U S A 114:12952–12957. doi:10.1073/pnas.1716002114. 29183968 PMC5724282

[18] Pourjaberi SNS, Terahara N, Namba K, Minamino T 2017 The role of a cytoplasmic loop of MotA in load-dependent assembly and disassembly dynamics of the MotA/B stator complex in the bacterial flagellar motor. Mol Microbiol 106:646–658. doi:10.1111/mmi.13843. 28925530

[19] Jenal U, White J, Shapiro L 1994 *Caulobacter* flagellar function, but not assembly, requires FliL, a non-polarly localized membrane protein present in all cell types. J Mol Biol 243:227–244. doi:10.1006/jmbi.1994.1650. 7932752

[20] Attmannspacher U, Scharf BE, Harshey RM 2008 FliL is essential for swarming: motor rotation in absence of FliL fractures the flagellar rod in swarmer cells of *Salmonella enterica*. Mol Microbiol 68:328–341. doi:10.1111/j.1365-2958.2008.06170.x. 18284590

[21] Suaste-Olmos F, Domenzain C, Mireles-Rodríguez JC, Poggio S, Osorio A, Dreyfus G, Camarena L 2010 The flagellar protein FliL is essential for swimming in *Rhodobacter sphaeroides*. J Bacteriol 192:6230–6239. doi:10.1128/JB.00655-10. 20889747 PMC2981197

[22] Motaleb MA, Pitzer JE, Sultan SZ, Liu J 2011 A novel gene inactivation system reveals altered periplasmic flagellar orientation in a *Borrelia burgdorferi fliL* mutant. J Bacteriol 193:3324–3331. doi:10.1128/JB.00202-11. 21441522 PMC3133274

[23] Cusick K, Lee YY, Youchak B, Belas R 2012 Perturbation of FliL interferes with *Proteus mirabilis* swarmer cell gene expression and differentiation. J Bacteriol 194:437–447. doi:10.1128/JB.05998-11. 22081397 PMC3256649

[24] Partridge JD, Nieto V, Harshey RM 2015 A new player at the flagellar motor: FliL controls both motor output and bias. mBio 6:e02367. doi:10.1128/mBio.02367-14. 25714720 PMC4358005

[25] Zhu S, Kumar A, Kojima S, Homma M 2015 FliL associates with the stator to support torque generation of the sodium-driven polar flagellar motor of *Vibrio*. Mol Microbiol 98:101–110. doi:10.1111/mmi.13103. 26103585

[26] Hall AN, Subramanian S, Oshiro RT, Canzoneri AK, Kearns DB 2017 SwrD (YlzI) promotes swarming in Bacillus subtilis by increasing power to flagellar motors. J Bacteriol 200:e00529-17. 29061663 10.1128/JB.00529-17 PMC5738730

[27] Fabela S, Domenzain C, De la Mora J, Osorio A, Ramirez-Cabrera V, Poggio S, Dreyfus G, Camarena L 2013 A distant homologue of the FlgT protein interacts with MotB and FliL and is essential for flagellar rotation in *Rhodobacter sphaeroides*. J Bacteriol 195:5285–5296. doi:10.1128/JB.00760-13. 24056105 PMC3837945

[28] Belas R, Suvanasuthi R 2005 The ability of *Proteus mirabilis* to sense surfaces and regulate virulence gene expression involves FliL, a flagellar basal body protein. J Bacteriol 187:6789–6803. doi:10.1128/JB.187.19.6789-6803.2005. 16166542 PMC1251568

[29] Chawla R, Ford KM, Lele PP 2017 Torque, but not FliL, regulates mechanosensitive flagellar motor-function. Sci Rep 7:5565. doi:10.1038/s41598-017-05521-8. 28717192 PMC5514156

[30] Kawagishi I, Maekawa Y, Atsumi T, Homma M, Imae Y 1995 Isolation of the polar and lateral flagellum-defective mutants in *Vibrio alginolyticus* and identification of their flagellar driving energy sources. J Bacteriol 177:5158–5160. doi:10.1128/jb.177.17.5158-5160.1995. 7665498 PMC177299

[31] Li N, Kojima S, Homma M 2011 Characterization of the periplasmic region of PomB, a Na^+^-driven flagellar stator protein in *Vibrio alginolyticus*. J Bacteriol 193:3773–3784. doi:10.1128/JB.00113-11. 21602350 PMC3147510

[32] Kim DE, Chivian D, Baker D 2004 Protein structure prediction and analysis using the Robetta server. Nucleic Acids Res 32:W526–W531. doi:10.1093/nar/gkh468. 15215442 PMC441606

[33] Brand J, Smith ESJ, Schwefel D, Lapatsina L, Poole K, Omerbašić D, Kozlenkov A, Behlke J, Lewin GR, Daumke O 2012 A stomatin dimer modulates the activity of acid-sensing ion channels. EMBO J 31:3635–3646. doi:10.1038/emboj.2012.203. 22850675 PMC3433786

[34] Browman DT, Hoegg MB, Robbins SM 2007 The SPFH domain-containing proteins: more than lipid raft markers. Trends Cell Biol 17:394–402. doi:10.1016/j.tcb.2007.06.005. 17766116

[35] Yokoyama H, Fujii S, Matsui I 2008 Crystal structure of a core domain of stomatin from *Pyrococcus horikoshii* Illustrates a novel trimeric and coiled-coil fold. J Mol Biol 376:868–878. doi:10.1016/j.jmb.2007.12.024. 18182167

[36] Tanaka H, Kato K, Yamashita E, Sumizawa T, Zhou Y, Yao M, Iwasaki K, Yoshimura M, Tsukihara T 2009 The structure of rat liver vault at 3.5 angstrom resolution. Science 323:384–388. doi:10.1126/science.1164975. 19150846

[37] Zhan H, Moore CS, Chen B, Zhou X, Ma X-M, Ijichi K, Bennett MVL, Li X-J, Crocker SJ, Wang Z-W 2012 Stomatin inhibits pannexin-1-mediated whole-cell currents by interacting with its carboxyl terminal. PLoS One 7:e39489. doi:10.1371/journal.pone.0039489. 22768083 PMC3387187

[38] Genetet S, Desrames A, Chouali Y, Ripoche P, Lopez C, Mouro-Chanteloup I 2017 Stomatin modulates the activity of the Anion Exchanger 1 (AE1, SLC4A1). Sci Rep 7:46170. doi:10.1038/srep46170. 28387307 PMC5383999

[39] Goodman MB, Ernstrom GG, Chelur DS, O'Hagan R, Yao CA, Chalfie M 2002 MEC-2 regulates *C. elegans* DEG/ENaC channels needed for mechanosensation. Nature 415:1039–1042. doi:10.1038/4151039a. 11875573

[40] Wetzel C, Hu J, Riethmacher D, Benckendorff A, Harder L, Eilers A, Moshourab R, Kozlenkov A, Labuz D, Caspani O, Erdmann B, Machelska H, Heppenstall PA, Lewin GR 2007 A stomatin-domain protein essential for touch sensation in the mouse. Nature 445:206–209. doi:10.1038/nature05394. 17167420

[41] Cheng YR, Jiang BY, Chen CC 2018 Acid-sensing ion channels: dual function proteins for chemo-sensing and mechano-sensing. J Biomed Sci 25:46. doi:10.1186/s12929-018-0448-y. 29793480 PMC5966886

[42] Kumar A, Isumi M, Sakuma M, Zhu S, Nishino Y, Onoue Y, Kojima S, Miyanoiri Y, Imada K, Homma M 2017 Biochemical characterization of the flagellar stator-associated inner membrane protein FliL from *Vibrio alginolyticus*. J Biochem 161:331–337. doi:10.1093/jb/mvw076. 28013221

[43] Snyers L, Umlauf E, Prohaska R 1998 Oligomeric nature of the integral membrane protein stomatin. J Biol Chem 273:17221–17226. doi:10.1074/jbc.273.27.17221. 9642292

[44] Tatsuta T, Model K, Langer T 2005 Formation of membrane-bound ring complexes by prohibitins in mitochondria. Mol Biol Cell 16:248–259. doi:10.1091/mbc.e04-09-0807. 15525670 PMC539169

[45] Takekawa N, Terahara N, Kato T, Gohara M, Mayanagi K, Hijikata A, Onoue Y, Kojima S, Shirai T, Namba K, Homma M 2016 The tetrameric MotA complex as the core of the flagellar motor stator from hyperthermophilic bacterium. Sci Rep 6:31526. doi:10.1038/srep31526. 27531865 PMC4987623

[46] Zhu S, Takao M, Li N, Sakuma M, Nishino Y, Homma M, Kojima S, Imada K 2014 Conformational change in the periplamic region of the flagellar stator coupled with the assembly around the rotor. Proc Natl Acad Sci U S A 111:13523–13528. doi:10.1073/pnas.1324201111. 25197056 PMC4169928

[47] Lin TS, Zhu S, Kojima S, Homma M, Lo CJ 2018 FliL association with flagellar stator in the sodium-driven *Vibrio* motor characterized by the fluorescent microscopy. Sci Rep 8:11172. doi:10.1038/s41598-018-29447-x. 30042401 PMC6057877

[48] Oliner JD, Kinzler KW, Vogelstein B 1993 *In vivo* cloning of PCR products in *E. coli*. Nucleic Acids Res 21:5192–5197. doi:10.1093/nar/21.22.5192. 8255776 PMC310636

[49] Le Roux F, Binesse J, Saulnier D, Mazel D 2007 Construction of a *Vibrio splendidus* mutant lacking the metalloprotease gene *vsm* by use of a novel counterselectable suicide vector. Appl Environ Microbiol 73:777–784. doi:10.1128/AEM.02147-06. 17122399 PMC1800747

[50] Imada K 2017 Design and preparation of the fragment proteins of the flagellar components suitable for X-ray crystal structure analysis. Methods Mol Biol 1593:97–103. doi:10.1007/978-1-4939-6927-2_7. 28389947

[51] Battye TG, Kontogiannis L, Johnson O, Powell HR, Leslie AGW 2011 iMOSFLM: a new graphical interface for diffraction-image processing with MOSFLM. Acta Crystallogr D Biol Crystallogr 67:271–281. doi:10.1107/S0907444910048675. 21460445 PMC3069742

[52] Winn MD, Ballard CC, Cowtan KD, Dodson EJ, Emsley P, Evans PR, Keegan RM, Krissinel EB, Leslie AGW, McCoy A, McNicholas SJ, Murshudov GN, Pannu NS, Potterton EA, Powell HR, Read RJ, Vagin A, Wilson KS 2011 Overview of the CCP4 suite and current developments. Acta Crystallogr D Biol Crystallogr 67:235–242. doi:10.1107/S0907444910045749. 21460441 PMC3069738

[53] Adams PD, Afonine PV, Bunkóczi G, Chen VB, Davis IW, Echols N, Headd JJ, Hung L-W, Kapral GJ, Grosse-Kunstleve RW, McCoy AJ, Moriarty NW, Oeffner R, Read RJ, Richardson DC, Richardson JS, Terwilliger TC, Zwart PH 2010 PHENIX: a comprehensive Python-based system for macromolecular structure solution. Acta Crystallogr D Biol Crystallogr 66:213–221. doi:10.1107/S0907444909052925. 20124702 PMC2815670

[54] Emsley P, Lohkamp B, Scott WG, Cowtan K 2010 Features and development of *Coot*. Acta Crystallogr D Biol Crystallogr 66:486–501. doi:10.1107/S0907444910007493. 20383002 PMC2852313

[55] Terashima H, Kojima S, Homma M 2010 Functional transfer of an essential aspartate for the ion-binding site in the stator proteins of the bacterial flagellar motor. J Mol Biol 397:689–696. doi:10.1016/j.jmb.2010.01.050. 20122938

[56] Karimova G, Pidoux J, Ullmann A, Ladant D 1998 A bacterial two-hybrid system based on a reconstituted signal transduction pathway. Proc Natl Acad Sci U S A 95:5752–5756. doi:10.1073/pnas.95.10.5752. 9576956 PMC20451

