# Supplementary material for: Structure of Vibrio FliL, a New Stomatin-like Protein That Assists the Bacterial Flagellar Motor Function
Source: mBio. 2019 Mar 19;10(2):e00292-19. doi: 10.1128/mBio.00292-19 (PMC6426602; doi:10.1128/mBio.00292-19)
Supplement: FIG S1 [file mBio.00292-19-sf001.pdf]

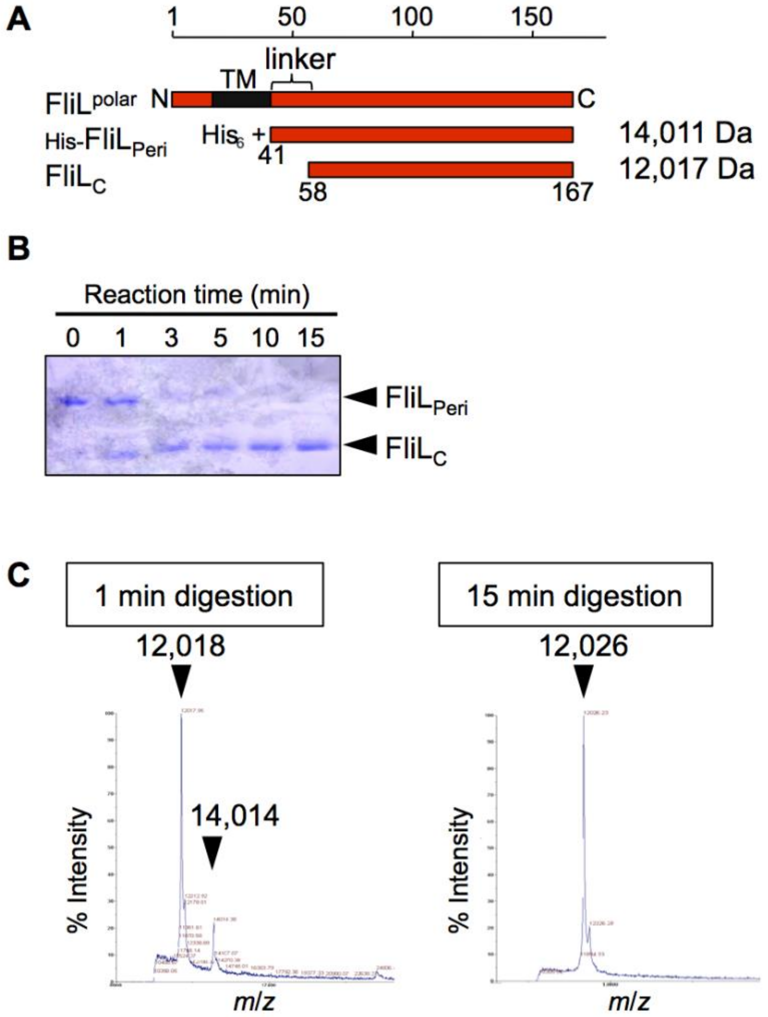

Figure S1. Limited proteolysis of the periplasmic region of FliL. (A) Molecular architecture of FliL and the N-terminally-truncated FliL fragments. The molecular weight of each fragment is indicated at right. (B) Time course profile of trypsin digestion of His-FliL$_{Peri}$ analyzed by SDS-PAGE. His-FliL$_{Peri}$ was digested into FliL$_C$. Trypsin was added one-hundredth the weight of His$_6$-FliL$_{Peri}$ protein and incubated at 27 °C. (C) Mass analysis of the trypsin digestion products of His-FliL$_{Peri}$. Undigested His-FliL$_{Peri}$ and a digested fragment were detected at m/z of ca. 14.0 and 12.0 kDa, respectively, using a MALDI-TOF (Matrix assisted laser desorption time of flight) mass spectrometer (Voyager-DE Pro; Applied Biosystems, Foster City, CA, USA).
