# Supplementary material for: Structure of Vibrio FliL, a New Stomatin-like Protein That Assists the Bacterial Flagellar Motor Function
Source: mBio. 2019 Mar 19;10(2):e00292-19. doi: 10.1128/mBio.00292-19 (PMC6426602; doi:10.1128/mBio.00292-19)
Supplement: FIG S2 [file mBio.00292-19-sf002.pdf]

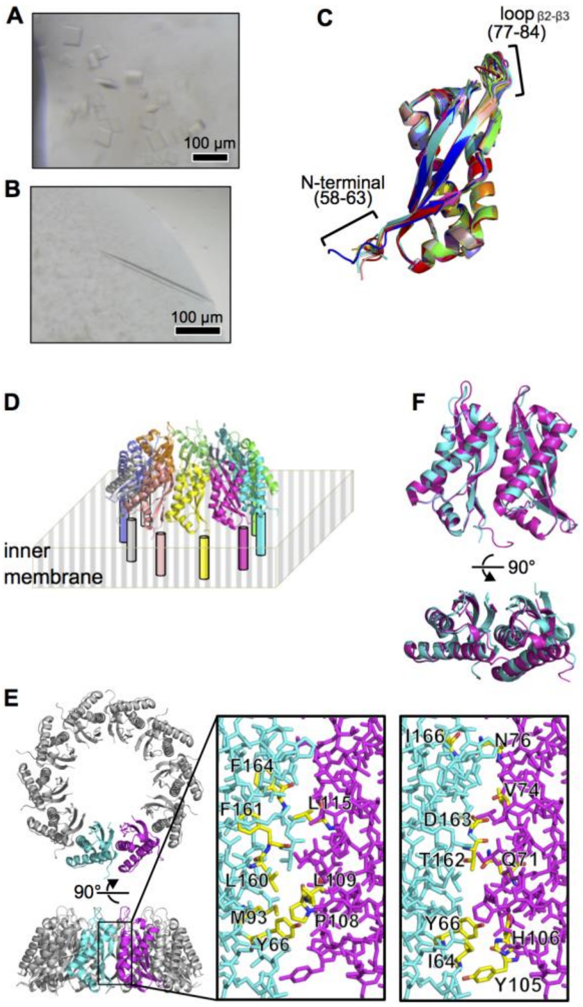

Figure S2. Comparison of the FliL structures in crystal. (A and B) Crystals of $FliL_{Peri}$ (A) and $FliL_{C}$ (B). (C) Superposition of 22 independent structures in the two crystal forms. (D) A model of a decameric ring of FliL on the inner membrane. The cylinders indicate the transmembrane helices of each FliL protomers. (E) Subunit interface of the FliL ring. Two adjacent subunits are colored in cyan and magenta. The hydrophobic residues (in the middle panel) and hydrophilic residues (in the right panel) contributing to the subunit interaction are labeled and highlighted in yellow. (F) Comparison of the molecular arrangement of the two adjacent subunits in the $FliL_{C}$ crystal (magenta) and in the $FliL_{Peri}$ crystal (cyan). The Cα atoms of the left subunits are superimposed.
