# Supplementary material for: Structure of Vibrio FliL, a New Stomatin-like Protein That Assists the Bacterial Flagellar Motor Function
Source: mBio. 2019 Mar 19;10(2):e00292-19. doi: 10.1128/mBio.00292-19 (PMC6426602; doi:10.1128/mBio.00292-19)
Supplement: TABLE S1 [file mBio.00292-19-st001.pdf]

Table S1. **Summary of the X-ray data statistics**

	$FliL_C$	$FliL_{Peri}$
Diffraction data statistics		
Space group	*P* 6_1	*P* 4_22_12
Cell dimensions (Å)	a = b = 104.2, c = 40.1	a = b = 162.2, c = 302.2
Wave length (Å)	1.000	1.000 or 1.0717
Resolution range (Å)	52.1 - 2.1 (2.2 - 2.1)	91.3 - 3.4 (3.5 - 3.4)
Number of observations	138,014 (11,233)	383,368 (27,942)
Number of unique reflections	14,756 (1,185)	54,933 (4,467)
Completeness (%)	99.9 (100.0)	98.1 (98.8)
Multiplicity	9.4 (9.5)	7.0 (6.3)
I/σ (*I*)	23.4 (5.3)	10.8 (5.0)
R_{merge} (%)	5.6 (43.5)	13.7 (32.5)
Refinement statistics		
No. of reflections working	14,737	54,840
No. of reflections test	1,468	2,715
R_w (%)	20.6 (25.0)	18.7 (25.6)
R_{free} (%)	25.8 (31.7)	24.5 (33.9)
Root mean square deviation		
Bond length (Å)	0.018	0.002
Bond angle (°)	1.593	0.452
B factors		
Protein atoms	43.1	51.5
Solvent atoms	45.2	-
Ramachandran plot (%)		
Most favored allowed	98.2	98.1
Allowed	1.9	2.0
Generously allowed	0.0	0.0
Disallowed	0.0	0.0
Number of protein atoms	1,693	16,591
Number of solvent atoms	159	-

The values in parentheses are for the highest-resolution shell.

$R_w = \Sigma \,||\, Fo \,|-|\, Fc \,||\, / \,\Sigma \,|\, Fo \,|$, $R_{free} = \Sigma \,||\, Fo \,|-|\, Fc \,||\, / \,\Sigma \,|\, Fo \,|$
