# Supplementary material for: Structure of Vibrio FliL, a New Stomatin-like Protein That Assists the Bacterial Flagellar Motor Function
Source: mBio. 2019 Mar 19;10(2):e00292-19. doi: 10.1128/mBio.00292-19 (PMC6426602; doi:10.1128/mBio.00292-19)
Supplement: FIG S3 [file mBio.00292-19-sf003.pdf]

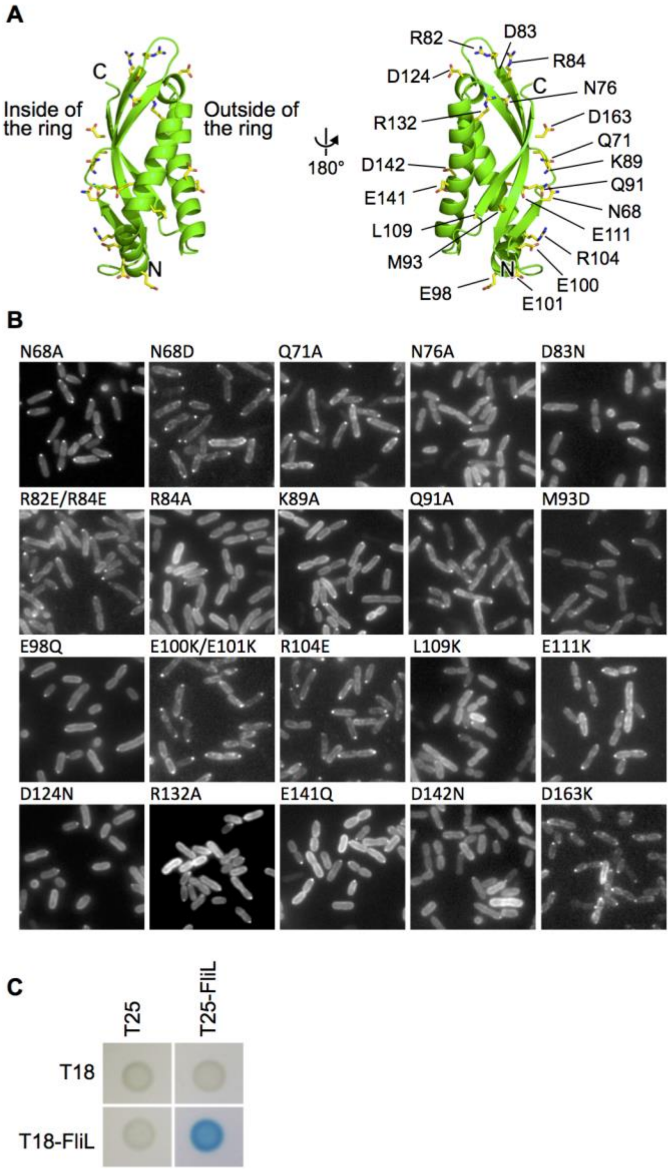

Figure S3. Fluorescence images of cells expressing various FliL mutant proteins labeled with GFP and results of bacterial two-hybrid assay. (A) Mapping of the mutations sites on the structure of FliL$_{\mathrm{C}}$. The mutation sites are indicated as yellow stick models. The orientation of the view is as in Fig. 2F. (B) Fluorescence images of cells expressing the mutant FliL proteins N-terminally labeled with GFP. (C) Bacterial two-hybrid assay. *E. coli* DHM1 cells expressing T18 and T25 alone or C-terminally fusions with FliL were spotted onto BacTH plate and incubated at 30 °C for about 24 h.
