# Supplementary material for: Structure of Vibrio FliL, a New Stomatin-like Protein That Assists the Bacterial Flagellar Motor Function
Source: mBio. 2019 Mar 19;10(2):e00292-19. doi: 10.1128/mBio.00292-19 (PMC6426602; doi:10.1128/mBio.00292-19)
Supplement: FIG S4 [file mBio.00292-19-sf004.pdf]

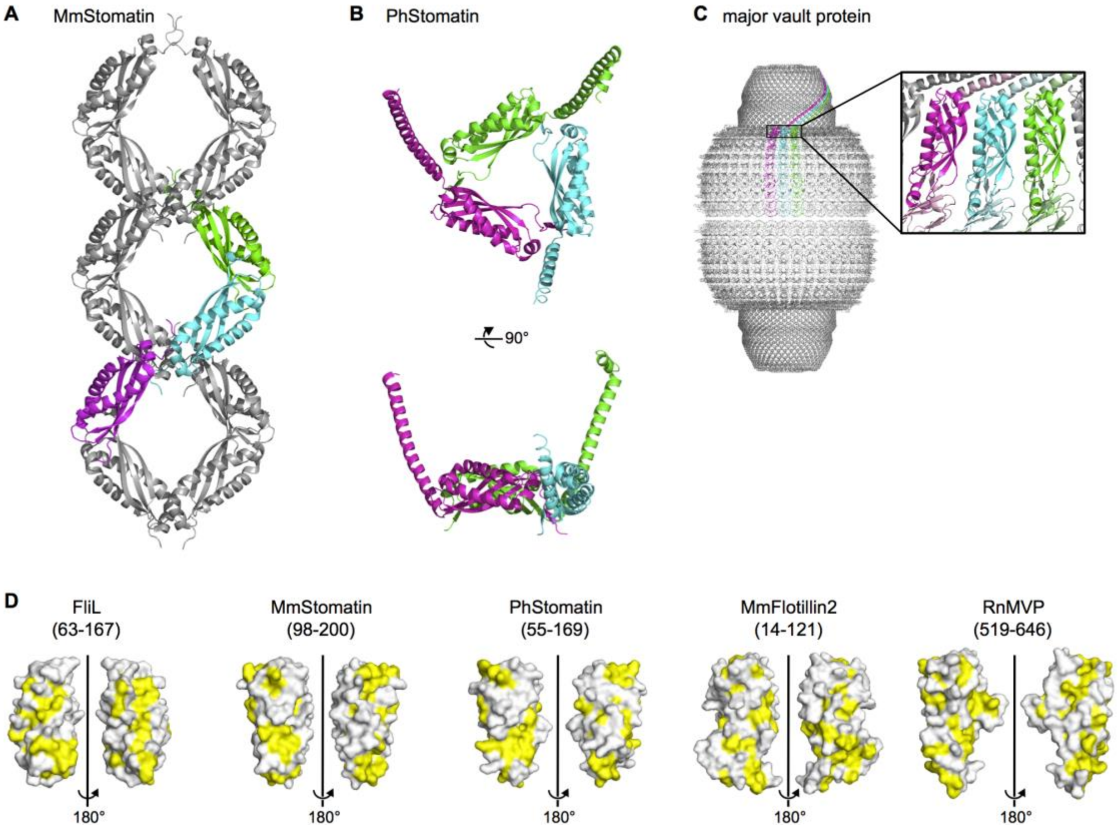

Figure S4. Multimer of the SPFH domains. (A) The helical complex of SPFH domain of MmStomatin in crystal form 1 (PDB ID: 4FVF). (B) The trimer of the SPFH domain of PhStomatin (PDB ID: 3BK6). (C) The SPFH domains of RnMVP in the vault complex (PDB ID: 4V60). Three adjacent subunits in the multimers are highlighted in green, cyan and magenta. (D) Distribution of the hydrophobic surface of the SPFH domains.
