# Supplementary material for: Structure of Vibrio FliL, a New Stomatin-like Protein That Assists the Bacterial Flagellar Motor Function
Source: mBio. 2019 Mar 19;10(2):e00292-19. doi: 10.1128/mBio.00292-19 (PMC6426602; doi:10.1128/mBio.00292-19)
Supplement: FIG S5 [file mBio.00292-19-sf005.pdf]

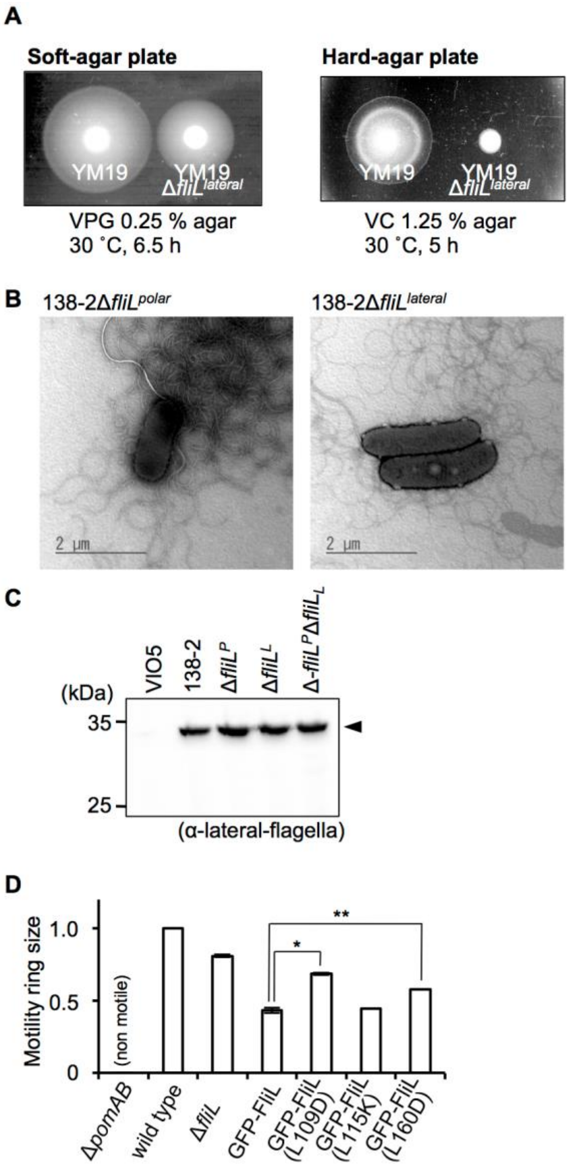

Figure S5. Effect of mutations on the motility and flagellation. (A) Representative images of the motility rings of YM19 and NMB342 strains. (B) Electron microscopic images of *fliL* deletion mutants of the wild-type strain 138-2. (C) Expression of lateral flagellin. Whole-cell extracts of VIO5, 138-2, NMB338, NMB339 and NMB340 strains were subjected to SDS-PAGE, followed by immunoblotting using anti-lateral flagellin antibody. (D) Effect of the N-terminal fusion of GFP to FliL on cell motility. Motility ring diameters relative to that of VIO5 strain are shown. Error bar, standard deviation. *, $p < 0.002$; **, $p < 0.02$, unpaired t-test. Overnight cultures of Δ*pomAB* strain, VIO5 strain, Δ*fliL* strain, and Δ*fliL* strain containing pZSW6 with/without mutations were spotted onto a VPG soft-agar plate containing 0.02% (w/v) arabinose and incubated at 30 °C for 3 h.
