# Supplementary material for: Structure of Vibrio FliL, a New Stomatin-like Protein That Assists the Bacterial Flagellar Motor Function
Source: mBio. 2019 Mar 19;10(2):e00292-19. doi: 10.1128/mBio.00292-19 (PMC6426602; doi:10.1128/mBio.00292-19)
Supplement: TABLE S2 [file mBio.00292-19-st002.pdf]

Table S2. Strains and plasmids used in this study.

Strain or plasmid	Description	Source or reference
E. coli strains		
DH5α	F^- λ^- *recA1 hsdR17 endA1 supE44 thi-1 relA1 gyrA96*Δ(*argF*-*lacZYA*) U169 φ80d*lacZ*ΔM15)<br>(Recipient for cloning experiments)	(57)
BL21(DE3)	F^- *ompT hsdS*(r_B^- m_B^-) *gal dcm* λ(DE3)<br>(Host for overproduction of proteins)	Novagen
β3914	β2163 *gyrA462 zei-298*::Tn*10* (Km^r Em^r Tc^r)<br>(Recipient for conjugational transfer of pSW7848)	(49)
DHM1	F^-, *cya-854, recA1, endA1, gyrA96 (Nal^r), thi1, hsdR17, spoT1, rfbD1, glnV44(AS).*<br>(Host for bacterial two-hybrid (BACTH) assay)	(58)
V. alginolyticus strains		
138-2	wild-type (Pof^+, Laf^+)	(59)
VIO5	Rif^r Pof^+ Laf^-	(60)
NMB191	VIO5 Δ*pomAB*	(61)
ZSW1	VIO5 Δ*fliL*^{P}	(25)
ZSW2	VIO5 Δ*pomAB* Δ*fliL*^{P}	(25)
YM19	138-2 Pof^-, Laf^+	(30)
NMB342	YM19Δ*fliL*^{L}	This study
NMB338	138-2 Δ*fliL*^{P}	This study
NMB339	138-2 Δ*fliL*^{L}	This study
NMB340	138-2 Δ*fliL*^{P}Δ*fliL*^{L}	
Plasmids		
pColdI	Amp^r P_{cspA} (Cold shock expression vector)	Takara
pZSW4	pColdI- *fliL*$^P_{Peri}$ (from Met40 to stop codon)	(25)
pSW7848	Suicide vector (oriVR6Kγ oriTRP4 *araC*-P_{BAD}-*ccdB*), Cm^r	(49)
pZSW9	pSW7848- Δ*fliL*^{L}	This study
pBAD33	Cm^r P_{BAD}	(62)
pZSW6	pBAD33- *his*$_6$-*egfp*-*fliL*^{P}	(25)

pZSW81	pBAD33/ *his*$_6$*-egfp-fliL*^{P} *pomApomB*	(47)
pZSW91	pBAD33- *his6-fliL*^{L}	This study
pEVOL-*p*BpF	Plasmid encodes an orthogonal *p*Bpa-specific suppressor tRNA/aminoacyl tRNA synthetase pair, Cm^r	(63)
pET15b	Amp^r P_{T7}	Novagen
pNT70	pET15b- *his*$_6$*-fliL*^{P} *pomApomB* (whose amber codon in *fliL*^{P} was replaced with ochre codon)	This study
pKT25	Km^r, T25-X (BACTH vector for protein fusion at the C-terminal end of T25 domain of CyaA)	(56)
pUT18C	Amp^r, T18-X (BACTH vector for protein fusion at the C-terminal end of T18 domain of CyaA)	(56)

Km^r, kanamycin resistant; Em^r, erythromycin resistant; Tc^r, tetracycline resistant; Rif^r, rifampicin resistant; Amp^r, ampicillin resistant; Cm^r, chloramphenicol resistant; Pof$^+$, normal polar flagellar formation; Laf$^+$, normal lateral flagellar formation; Pof$^-$, defective in polar flagellar formation; Laf$^-$, defective in lateral flagellar formation; *fliL*^{P}, polar *fliL*; *fliL*^{L}, lateral *fliL*; P_{cspA}, cold-shock promoter; P_{BAD}, arabinose promoter. pEVOL-*p*BpF was a gift from Prof. Peter G. Schultz (Addgene plasmid #31190; http://n2t.net/addgene:31190; RRID:Addgene_31190).

57. Grant SG, Jessee J, Bloom FR, Hanahan D (1990) Differential plasmid rescue from transgenic mouse DNAs into *Escherichia coli* methylation-restriction mutants. *Proc Natl Acad Sci USA* **87**:4645-4649.
58. Karimova G, Dautin N, Ladant D (2005) Interaction network among *Escherichia coli* membrane proteins involved in cell division as revealed by bacterial two-hybrid analysis *J Bacteriol.* **187**:2233-2243.
59. Tokuda H, et al. (1988) Roles of the respiratory Na^+ pump in bioenergetics of *Vibrio alginolyticus*. *J Biochem* **103**:650-655.
60. Okunishi I, Kawagishi I, Homma M (1996) Cloning and characterization of *motY*, a gene coding for a component of the sodium-driven flagellar motor in *Vibrio alginolyticus*. *J Bacteriol* **178**:2409-2415.
61. Yorimitsu T, Sato K, Asai Y, Kawagishi I, Homma M (1999) Functional interaction between PomA and PomB, the Na^+-driven flagellar motor components of *Vibrio alginolyticus*. *J Bacteriol* **181**: 5103-5106.

62. Guzman LM, Belin D, Carson MJ, Beckwith J (1995) Tight regulation, modulation, and high-level expression by vectors containing the arabinose P_{BAD} promoter. *J Bacteriol* **177**:4121-4130.
63. Chin JW, Martin AB, King DS, Wang L, Schultz PG (2002) Addition of a photocrosslinking amino acid to the genetic code of *Escherichia coli*. *Proc Natl Acad Sci U S A*. **99**:11020-11024.
